# Effects of Moderate- and High-Intensity Chronic Exercise on the Adiponectin Levels in Slow-Twitch and Fast-Twitch Muscles in Rats

**DOI:** 10.3390/medicina55060291

**Published:** 2019-06-19

**Authors:** Alberto Jiménez-Maldonado, Adolfo Virgen-Ortiz, Mónica Lemus, Elena Castro-Rodríguez, Joel Cerna-Cortés, Jesús Muñiz, Sergio Montero, Elena Roces

**Affiliations:** 1 Department of Neuroencocrinology, University Center of Biomedical Research, Colima University, Colima 28045, Mexico; jimenez.alberto86@uabc.edu.mx (A.J.-M.); avirgen@ucol.mx (A.V.-O.); mlv@ucol.mx (M.L.); ecastro@ucol.mx (E.C.-R.); 2 Sport Faculty, Campus Ensenada, Baja California Autonomic University, Baja California 22890, Mexico; 3 Medicine Faculty, Colima University, Colima 28040 Mexico; joelcerna@ucol.mx; 4 Institute of Cancerology, Colima State Health Services, Colima 28060, Mexico; jesusmunizmurguia@gmail.com

**Keywords:** adiponectin, chronic exercise, high-intensity training, moderate-intensity training, slow and fast muscles

## Abstract

*Background and objectives*: Adipose tissue and skeletal muscle secrete adiponectin, a hormone abundantly secreted by adipocytes, that through the adiponectin receptor, regulate glucose and lipid metabolism. Adiponectin appears to protect skeletal muscles from inflammatory damage induced by oxidative stress. It has been suggested that decreased adiponectin levels could be associated with pathologic conditions, including obesity and diabetes. Furthermore, some studies suggest that exercise could have a beneficial effect by increasing adiponectin levels, but this observation remains controversial. It is also unknown if physical exercise modifies adiponectin expression in skeletal muscles. The aim of this study was to investigate the effect of chronic exercise on serum adiponectin and adiponectin expression in slow-twitch (soleus) and fast-twitch (plantaris) muscles in healthy rats. *Materials and methods*: Two-month-old male Wistar rats were randomly divided into three groups with *n* = 6 in each group: control (C), moderate-intensity training (MIT), and high-intensity training (HIT). The rats were conditioned to run on a treadmill for the 8-week period. Forty-eight hours after the last session, blood samples were collected for adiponectin measurements and total RNA was isolated from plantaris and soleus muscles to measure by RT-qPCR adiponectin receptor 1 and adiponectin mRNA expression level. *Results*: MIT and HIT groups had reduced adiponectin protein levels in serum and the plantaris muscle, but not changes in adiponectin protein were observed in the soleus muscle. No significant differences in Adiponectin receptor 1 (AdipoR1) gene expression were observed following intense or moderate exercise in either muscle group studied. *Conclusions:* Our study shows that decreasing levels of circulating adiponectin is a result of physical exercise and should not be generalized as a predictive marker of disease.

## 1. Introduction

Adiponectin, also known as Adipoq is an adipocyte-derived factor [1]. Through adiponectin receptor activation (Adipor1 and Adipor2), this adipokine regulates glucose and lipid metabolism and has anti-inflammatory properties [2,3,4]. The first reports stated that adiponectin synthesis and its release took place only by the adipose tissue [5]. However, more recent work has shown that other peripheral organs, such as skeletal muscles, secrete adiponectin [4,6,7]. Since then, authors have tried to identify the role that adiponectin plays in skeletal muscle cells. Adiponectin over-expression, using molecular engineering techniques, increases insulin sensitivity and improves muscle performance [8,9]. Furthermore, adiponectin has anti-atherogenic and anti-inflammatory properties, and protects the skeletal muscle from oxidative stress, and apoptosis induced by a high caloric diet (Western diet) [10]. Adiponectin also improves muscular physiology in diseases, such as Duchenne muscular dystrophy [11]. In addition, adiponectin has also a positive role on the muscle mitochondrial mass [12]. Adiponectin on skeletal muscles have been intensively studied, but the origin of circulating adiponectin that acts on skeletal muscle cells remains unclear.

Previous authors report that mechanical loading modifies adiponectin expression in slow-twitch muscle [7], but it remains unknown if physical exercise affects adiponectin expression in skeletal muscle. The focus of the present work is to study the effect of training intensity on adiponectin expression in slow-twitch and fast-twitch muscles in young healthy rats. Since muscle phenotype affects protein and gene expression related to neuromuscular transmission, glucose metabolism, and muscle growth after chronic exercise [13,14] we hypothesized that muscle phenotype plays a role in adiponectin expression in a healthy state after chronic physical exercise.

## 2. Methods

### 2.1. Ethics

The experiments were performed in accordance with the United States National Institutes of Health Guidelines for the Care and Use of Laboratory Animals. The Bioethics and Biosecurity Committee of the School of Medicine and the CUIB of Colima University (No. 2012-05) approved on Second/February/2012 the animal studies and experimental procedures for the present study.

We confirm that we have read the Journal’s position on issues involved in ethical publication and affirm that this report is consistent with those guidelines.

### 2.2. Animals and Experimental Protocol

Eighteen 7-week-old lean male Wistar rats, individually housed in polyethylene cages were used. Temperature was controlled at 22–24 °C, with a 12-h light/dark cycle. The rats were given free access to food (Teklad Global Diet: protein/fat/fiber 18.0%/5.0%/5.0%, respectively) and water, and were weighed each week. It was consistent with the 3Rs principles by achieving significant reductions in the number of animals used, decreased restraints and animal stress, and improved data quality [15]. The animals were randomly divided into the following groups: control sedentary-C (*n* = 6); moderate-intensity training (MIT) (*n* = 6); high-intensity training (HIT) (*n* = 6). The training protocol was performed over an 8-week period, and the exercise sessions were carried out between 9 a.m and 12 p.m.

### 2.3. Training Schedule

The rats were exercised following a protocol described by Wisloff et al. [16] with some modifications. Briefly, before the exercise program, all animals underwent a preconditioning running regime for a week that consisted of 30 min of daily running at a speed of 15 m/min on a rat treadmill (Modular Treadmill Simplex, Mod. 42528; Columbus Instruments, Columbus, OH, USA). An electrified grid (0.6 mA intensity) placed behind the belt of the treadmill induced running. The rats that did not run regularly were excluded from the training protocol. The rats were subjected to a workload on the treadmill with progressive increases in tilt and speed. Initially, rats were exposed to a warming period of 10 min at 0% inclination and 18 m/min, then in five stages of 2 min, the inclination and speed were increased to reach 25% and 22 or 28 m/min to perform a training period of 30 min. The last 10 min corresponded to the cooldown at 0% inclination and 18 m/min. The total duration of training was 60 min. These protocols were applied 3 days (Monday, Wednesday, Friday) a week for 8 weeks. The power (P) developed at 22 m/min, 25% inclination, was 1.8 kpm/min, with training at 28 m/min, 25% inclination, the P performed was 2.3 kpm/min. Leandro et al. (2007) [17] reported that rats reached 100% of maximum oxygen consumption (VO_2_max) performing a run at a speed of 18 m/min and a slope of 10%, challenge equivalent to a P of 0.5 kpm/min. Wisløff et al. (2001) [16] recorded that the rat reached 100% of VO2max when ran at 39 m/min, with a 25% incline, developing at P of 4.5 kpm·min^−1^. In addition, Garekani et al. (2011) [18] trained Wistar rats with the following protocols: high-intensity (HI: 34 m/min ~85% VO2 max, P: 0.629 kpm/min), moderate-intensity (MI: 28 m/min ~75% VO2 max, P: 0.518 kpm/min), low-intensity (LI: 20m/min ~55% VO2 max, P: 0.37 kpm/min). Our calculations were made based on these previous studies [16,17,18] and estimated that our training regime result ~60% or 80% of the maximal oxygen consumption for MIT and HIT groups respectively.

### 2.4. Skeletal Muscle Sample

The soleus and plantaris muscles were removed following dissection from the surrounding tissue in anesthetized rats. Soleus and plantaris were dissected from both the right and left hind limbs in all animals. Muscle samples were stored at -76 °C until processed by the ELISA assay and to measure mRNA level by real-time RT-qPCR.

### 2.5. Measurement of Adiponectin Levels in Serum and Muscle Tissue

Forty-eight hours after the final training session and before the surgical procedures, all the rats were lightly anesthetized with sodium pentobarbital (2.5 mg/100 g body weight, i.p.), and 5 mL of venous blood was collected via cardiac puncture using BD Vacutainer tubes (Becton Dickinson, Mexico City, Mexico). The blood samples were left to clot at room temperature for 30 min, centrifuged at 3000× *g* for 15 min at 4 °C, and serum samples were transferred in separate tubes and stored at −76 °C. The soleus and plantaris muscle samples were homogenized in lysis buffer (25 mM Tris-HCl pH 6.8, 2% SDS, 5 mM EDTA, 0.1% Aprotinin, 0.1% Leupeptin, 0.035% Pepstatin A, 8.5% PMSF) and then centrifuged as above. The supernatant was collected in conic tubes and stored at −76 °C until assayed. The amount of total protein was quantified in the muscle homogenates using the Bradford method. Adiponectin levels were then measured in serum and muscle homogenate samples with the Rat Adiponectin ELISA Kit, according to the manufacturer’s instructions (Cat. # EZRADP-62K, EMD Millipore, Merck, Mexico City, Mex). The analysis was carried out using serum and muscle homogenate samples diluted to 1:500. Absorbance was measured at 450 nm (Multiskan Ascent microplate reader, Thermo Electron Corporation, Vantaa, Finland) within the first 30 min after the stop solution was added. Samples were tested in duplicate, and the mean absorbance was calculated. The sensitivity limit of this assay was 0.4 ng/mL and quality control verified with the standards included in the kit. The adiponectin levels are expressed in concentration units for the serum samples, and in concentration unit/amount of total protein for the muscle samples.

### 2.6. Adiponectin Receptor 1 and Adiponectin mRNA Expression in Muscle Samples Based on RT-qPCR

Muscle samples were thawed, weighed, and immersed in TRIzol. RNA isolation was performed as previously described [14]. The RNA pellet was resuspended in 50 µL of 0.01% diethyl pyrocarbonate bi-distilled-in water (DEPC-ddH2O). The concentration and purity of RNA were analyzed using the Biophotometer (Eppendorf, Hamburg, Germany), and optical densities at 260/280 and 260/230 nm. For each sample, 100 ng of RNA was subjected to reverse-transcription polymerase chain reaction (RT-PCR) using the LightCycler RNA Master SYBR Green I kit (Roche, Nutley, NJ, USA). The following primers for the Adipor1, Adipoq, and β-actin (Actb) genes were used to amplify a 225 bp fragment of the Adipor1 mRNA: sense: 5′-CAAGGCTGAAGAAGAACAAGC-3′ and antisense: 5′-AAGGAGGGCATAGGTGGTCT-3′. To amplify a 165 bp fragment of the Actb mRNA, the following primers were used: sense: 5′-TGTCACCAACTGGGACGATA-3′ and antisense: 5′-GGGGTGTTGAAGGTCTCAAA-3′ and to amplify a 245 bp fragment of the Adipoq gene the following primers were used: sense 5′-CACTGTCCCCAATGTTCCCA-3′ and antisense 5′-TCCAGATGGAGGAGCATGGA-3′. Standard curve experiments were carried out to determine the efficiency of amplification for each primer set. Each reaction was made in duplicate. The quantification cycle (Cq) values for the Adipor1 gene were normalized to the values for the endogenous housekeeping gene Actb (ΔCq) and used to obtain the relative gene expression according to the formula 2 − ΔΔCq, where ΔΔCq = (Cq Adipor1 − Cq Actb) problem tissue −(Cq Adipor1 − Cq Actb) control tissue.

### 2.7. Data Presentation and Statistical Analysis

The results are presented as means ± S.E.M. from the indicated numbers of the experiments. All statistical analyses were done using the SPSS 17.0 statistical software package. Comparisons between the experiments were made using one-way ANOVA, and the Tukey’s post hoc test. Differences were considered significant at *p* < 0.05. The coefficient of variation (CV) was calculated as their standard deviation divided by their mean. The graphs were prepared with GraphPad Prism software.

## 3. Results

### 3.1. Effect of Chronic Exercise on Body Weight and Serum Adiponectin

**Figure 1:**
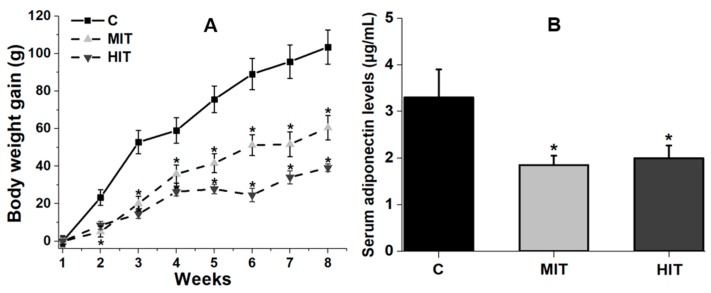
Effects of an 8-week treadmill exercise on body weight gain and systemic adiponectin in adult rats. (**A**) Time-course in the body weight gain through the training protocol. (**B**) Adiponectin levels in: serum for control, sedentary condition C, moderate-intensity training MIT, and high-intensity training HIT groups. * *p* < 0.05 vs. C. Values are means ± SEM. One way ANOVA and Tukey’s post hoc.

At the beginning of the experiments, the average body weight was the same in the three groups: C, 237 ± 13.02 g; MIT, 239.4 ± 4.8 g; and HIT, 247 ± 6.3 g. At the end of the 8th week, the body weight gain in C group was 103.5 ± 9.1 (CV = 19%) g, while in the MIT group gain was 60.5 ± 6.5 g (CV = 21%) (*p* = 0.07 vs. C) and in HIT was 39.2 ± 2.1 g (CV = 10%) (*p* = 0.009 vs. C) (Figure 1A). Regarding serum adiponectin concentration, we found that in C group was 3.25 ± 0.44 μg/mL (CV = 27%), in the MIT group it was 1.9 ± 0.15 μg/mL (*p* = 0.01 vs. C) (CV = 15%), and in the HIT group it was 2.04 ± 0.2 μg/mL (*p* = 0.02 vs. C) (CV = 19%). Statistical comparison between MIT and HIT groups with C reveals that the trained groups have a significantly lower serum adiponectin concentration, as was previously shown [19] (Figure 1B).

### 3.2. Effect of Training Intensity on Adiponectin Protein Content and Adiponectin mRNA Expression in Fast-Twitch Muscle

**Figure 2:**
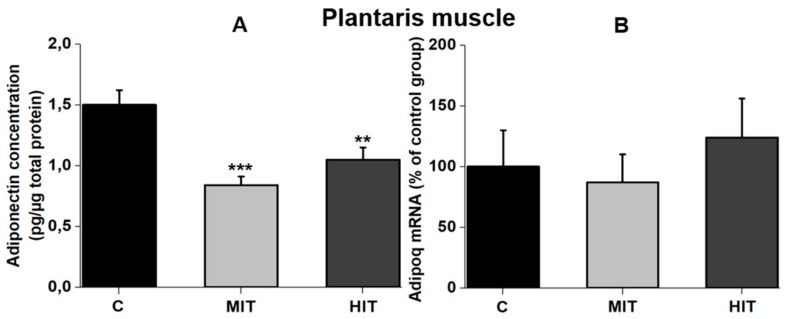
Adiponectin levels in fast-twitch muscle (plantaris) in adult rats. (**A**) Adiponectin protein content in the plantaris muscle. (**B**) Adipoq mRNA levels in the control group, after moderate- and high-intensity training. Control conditions C, moderate-intensity training MIT, and high-intensity training HIT. *** *p* = 0.00005 vs. C. ** *p* = 0.001 vs. C. Values are the means ± SEM. One way ANOVA and Tukey’s post hoc.

Moderate- and high-intensity training significantly reduced the adiponectin concentration in the plantaris muscle, compared with the sedentary conditions. Adiponectin concentration for C group was 1.5 ± 0.08 pg/μg total protein (CV = 10%). In the MIT group, adiponectin levels were 0.9 ± 0.03 pg/μg total protein (*** *p* = 0.00005 vs. C) (CV = 6%), whereas adiponectin concentration for the HIT group was 1.06 ± 0.06 pg/μg total protein (** *p* = 0.001 vs. C) (CV = 11%) (Figure 2A). To identify whether the lower protein levels for adiponectin in the plantaris muscle, after chronic exercise, was a result of a negative effect on Adipoq expression, we determined the adiponectin mRNA levels (percentage from group C in the plantaris muscle). The expression of adiponectin mRNA was not modified by chronic exercise. The Adipoq mRNA levels for the C and MIT groups were 100 ± 30 (CV = 60%) and 90 ± 20 (CV = 44%) (*p* = 0.2 vs. C), respectively, while in the HIT group, the adiponectin mRNA levels were 125 ± 30 (CV = 48%) (*p* = 0.5 vs. C) (Figure 2B).

### 3.3. Effect of Training Intensity on Adiponectin Protein Content and Adiponectin mRNA Expression in Slow-Twitch Muscle

**Figure 3:**
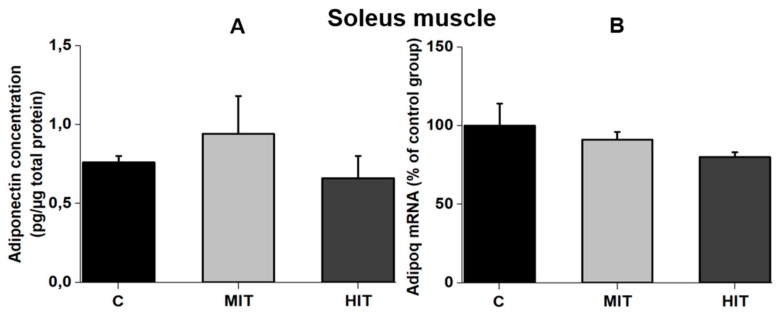
Adiponectin levels in a slow-twitch muscle (soleus) in adult rats. (**A**) Adiponectin protein content in the soleus muscle. (**B**) Adipoq mRNA levels in control group C, and after moderate-training MIT and high-intensity training HIT groups. Values are means ± SEM.

In contrast to the fast-twitch muscle, chronic exercise did not change the adiponectin level in the slow-twitch muscle (soleus). Adiponectin concentration for the C and MIT groups (plantaris) was 0.78 ± 0.04 pg/μg total protein (CV = 10%) and 0.96 ± 0.26 pg/μg total protein (CV = 56%) (*p* = 0.09 vs. C), respectively (Figure 3A), and the adiponectin level for the HIT group was 0.70 ± 0.14 pg/μg total protein (CV = 40%) (*p* = 0.6 vs. C) (Figure 3A). Coupled with the protein levels, chronic exercise did not affect the expression of Adipoq mRNA in the soleus muscle. Adipoq mRNA levels (percentage from C group in the soleus muscle), in C, MIT, and HIT groups they were 100 ± 15.9 (CV = 32%), 94 ± 5.6 (CV = 12%) (*p* = 0.2 vs. C), and 87.3 ± 2.8 (CV = 7%) (*p* = 0.1 vs. C), respectively (Figure 3B).

### 3.4. Adipor1 mRNA Expression after Chronic Exercise in Fast-Twitch and Slow-Twitch Muscles

**Figure 4:**
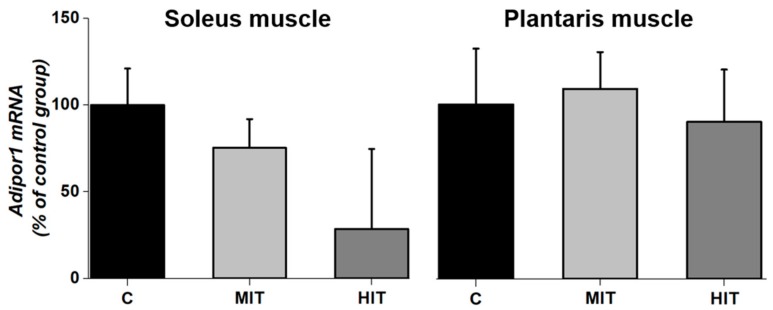
Effects of an 8-week treadmill exercise on Adipor1 mRNA levels in the plantaris (fast-twitch) and soleus (slow-twitch) muscles in adult rats. C, Control, MIT, moderate-intensity exercised rats; HIT, high-intensity exercised rats. Values are means ± SEM.

Chronic exercise did not have effect on Adipor1 mRNA levels (percentage from group C) in fast-twitch and slow-twitch muscles. In the C and MIT groups, mRNA Adipor1 expression was 100 ± 22% (CV = 24%) and 75 ± 3% (CV = 8%), respectively (*p* = 0.2 vs. C) whereas for HIT group, Adipor1 mRNA expression was 30 ± 40% (CV = 260%) (*p* = 0.5 vs. C) (Figure 4, soleus muscle). Similarly, Adipor1 mRNA expression in the plantaris muscle for C and MIT groups was 100.0 ± 30% (CV = 62%) and 107.0 ± 20% (CV = 39%), respectively. As occurred with moderate training, high-intensity training did not modify Adipor1 expression. The value for HIT was 87 ± 30% (CV = 71%) (Figure 4, plantaris muscle). 

## 4. Discussion

It is widely generalized in the literature that a reduction in adiponectin levels is associated with pathologic conditions, such as obesity [19,20], or diabetes [21,22]. Furthermore, some authors have suggested that exercise could have a beneficial effect by increasing adiponectin levels [23,24]. We think that rats doing intense or moderate exercise for eight weeks have reduced adiponectin protein levels in serum and plantaris muscle. In slow-twitch muscle (soleus), adiponectin concentration was not altered by moderate- or high-intensity training. Our study suggests that a decrease in adiponectin protein concentration in the fast-twitch muscle (plantaris) after moderate- or high-intensity exercise could be due to a decrease in adiponectin secretion, since Adipoq was not affected. By the contrary, in the slow-twitch muscle (soleus) adiponectin concentration did not modify after moderate- or high-intensity training. The results obtained in the present study in juvenile healthy rats showed a significant increase in body weight (BW) gain after MIT or HIT exercise, but this increase was always lower when compared to control rats without exercise. In the same way, serum adiponectin, as well as the plantaris muscle (fast-twitch) also were significantly lower when compared to the control group. By the contrary, the soleus muscle (slow-twitch) did not change significantly after MIT or HIT chronic exercise. Adipor1 expression in both, fast-twitch and slow-twitch muscles was not modified by chronic exercise.

It is known that adiponectin is mainly expressed in adipose tissue, but a lower expression has also been reported for other tissues including skeletal muscle, cardiomyocytes, and liver [25]. The lower velocity training used in our study (22 and 28 m/min) was closer to that used by Gómez-Merino [23] compared to other studies that used repetitions at 65–75% from the maximal repetition test, reporting no effect of aerobic exercise training on BW in healthy rats [26]. The lower adiponectin concentration obtained in serum for MIT and HIT groups (Figure 1B) is consistent with previous data [27] and indicate a scarce negative effect of endurance exercise on serum adiponectin levels in healthy subjects or athletes [28]. However, these observations do not agree with another study using healthy rats [18] in which moderate- and high-intensity exercise were reported to increase plasma adiponectin. In humans, adiponectin serum levels significantly increased in obese children after physical exercise protocol [29]; but other papers report lower levels of adiponectin in obese children compared with normal weight children [30]. A recent study indicated that skeletal muscle adiponectin induction depends on diet, muscle type/activity, and exercise modality [31]. We assume that in our trained rats there was also a decrease in the adipose tissue, reflected by the lower BW gain than control rats. Reduced levels of adipose tissue could explain the lower adiponectin serum levels [27]. This could also explain our data with reduced adiponectin levels in MIT and HIT groups in fast-twitch plantaris muscle (Figure 2A), without affecting Adipoq expression (Figure 2B). By the contrary, in the slow-twitch muscle, neither moderate- nor high-intensity training affected adiponectin concentration or transcriptional expression (Figure 3A,B). The higher oxidative capacity present in the soleus muscle, and the low intramyocellular lipid (IMCL) levels [30,32] could explain why compared to the plantaris, adiponectin levels were not affected by exercise in the slow-twitch muscle.

Adipor1 expression in both, fast-twitch and slow-twitch, muscles were not modified by chronic exercise (24 training sessions) (Figure 4), consistent with another study also using healthy rats [3]. Although exercise conditions may regulate adiponectin receptor mRNA expression in tissues and might cause some increases in adiponectin levels [3], time is essential to modify Adipor1 and could regulate mitochondrial mass in skeletal muscle under healthy conditions [12]. However, some studies do not find an effect of adiponectin signaling in the regulation of mitochondrial enzymes [21,32]. Our data, and results from previous works [21,32], suggest that adiponectin receptor (AdipoR1) is not likely to play a major role in muscle metabolic state during endurance training healthy rats. Our study shows that adiponectin response to exercise differs depending on muscle type (fast-twitch vs. low-twitch). A review by Simpson and Singh (2008) [24] finds that only three of eight studies show an increase in adiponectin levels following exercise. Despite the benefits of adiponectin to increase insulin sensitivity, the function of different isoforms of adiponectin is not clear, and exercise seems to differentially regulate each of them. Adiponectin levels might be influenced by multiple factors (species, sex, pathological condition, duration and type of exercise). For example, in healthy young subjects, both acute and chronic aerobic exercise did not alter plasma level of adiponectin [33]. Our paper showed that muscle phenotype plays a role in response to chronic exercise with respect to adiponectin levels.

## 5. Conclusions

Our study shows a decrease in adiponectin protein concentration in the fast-twitch muscle (plantaris) as well as serum adiponectin levels after moderate- or high-intensity exercise. Decreasing levels of circulating adiponectin should not be generalized as a predictive marker of disease.

## References

[1] Fu Y., Luo N., Klein R.L., Garvey W.T. (2005). Adiponectin promotes adipocyte differentiation, insulin sensitivity, and lipid accumulation. J. Lipid. Res..

[2] Yamauchi T., Kamon J., Ito Y., Tsuchida A., Yokomizo T., Kita S., Sugiyama T., Miyagishi M., Hara K., Tsunoda M. (2003). Cloning of adiponectin receptors that mediate antidiabetic metabolic effects. Nature.

[3] Zeng Q., Isobe K., Fu L., Ohkoshi N., Ohmori H., Takekoshi K., Kawakami Y. (2007). Effects of exercise on adiponectin and adiponectin receptor levels in rats. Life Sci..

[4] Van Berendoncks A.M., Garnier A., Beckers P., Hoymans V.Y., Possemiers N., Fortin D., Martinet W., Van Hoof V., Vrints C.J., Ventura–Clapier R. (2010). Functional adiponectin resistance at the level of the skeletal muscle in mild to moderate chronic heart failure. Circ. Heart Fail..

[5] Hu E., Liang P., Spiegelman B.M. (1996). AdipoQ is a novel adipose-specific gene dysregulated in obesity. J. Biol. Chem..

[6] Krause M.P., Liu Y., Vu V., Chan L., Xu A., Riddell M.C., Sweeney G., Hawke T.J. (2008). Adiponectin is expressed by skeletal muscle fibers and influences muscle phenotype and function. Am. J. Physiol. Cell. Physiol..

[7] Goto A., Ohno Y., Ikuta A., Suzuki M., Ohira T., Egawa T., Sugiura T., Yoshioka T., Ohira Y., Goto K. (2013). Up–regulation of adiponectin expression in antigravitational soleus muscle in response to unloading followed by reloading, and functional overloading in mice. PLoS ONE.

[8] Satoh H., Nguyen M.T., Trujillo M., Imamura T., Usui I., Scherer P.E., Olefsky J.M. (2005). Adenovirus–mediated adiponectin expression augments skeletal muscle insulin sensitivity in male Wistar rats. Diabetes.

[9] Safwat Y., Yassin N., Gamal E.l., Din M., Kassem L. (2013). Modulation of skeletal muscle performance and SERCA by exercise and adiponectin gene therapy in insulin–resistant rat. DNA Cell Biol..

[10] Jortay J., Senou M., Abou–Samra M., Noel L., Robert A., Many M.C., Brichard S.M. (2012). Adiponectin and skeletal muscle: pathophysiological implications in metabolic stress. Am. J. Pathol..

[11] Jortay J., Senou M., Abou–Samra M., Noel L., Robert A., Many M.C., Brichard S.M. (2015). Involvement of adiponectin in the pathogenesis of dystrophinopathy. Skelet Muscle.

[12] Civitarese A.E., Ukropcova B., Carling S., Hulver M., DeFronzo R.A., Mandarino L., Ravussin E., Smith S.R. (2006). Role of adiponectin in human skeletal muscle bioenergetics. Cell Metab..

[13] Leiter J.R., Peeler J., Anderson J.E. (2011). Exercise–induced muscle growth is muscle-specific and age–dependent. Muscle Nerve.

[14] Jiménez–Maldonado A., Cerna–Cortés J., Castro–Rodríguez E.M., Montero S.A., Muñiz J., Rodríguez–Hernández A., Lemus M., De Álvarez–Buylla E.R. (2016). Effects of moderate and high–intensity chronic exercise on brain–derived neurotrophic factor expression in fast and slow muscles. Muscle Nerve.

[15] Guhad F. (2005). Introduction to the 3Rs (Refinement, Reduction and Replacement). Contemp. Top. Lab. Anim. Sci..

[16] Wisløff U., Helgerud J., Kemi O.J., Ellingsen O. (2001). Intensity–controlled treadmill running in rats: VO(2 max) and cardiac hypertrophy. Am. J. Physio. Heart Circ. Physiol..

[17] Leandro C.G., Levada A.C., Hirabara F.M., Manhães–de–Castro R., De–Castro C.B., Curi R., Pithon–Curi T.C. (2007). A programof moderate physical training for Wistar rats based on maximal oxygen consumption. J. Strength Cond. Res..

[18] Garekani E.T., Mohebbi H., Kraemer R.R., Fathi R. (2011). Exercise training intensity/volume affects plasma and tissue adiponectin concentrations in the male rat. Peptides.

[19] Ritchie I.R., MacDonald T.L., Wright D.C., Dyck D.J. (2014). Adiponectin is sufficient, but not required, for exercise-induced increases in the expression of skeletal muscle mitochondrial enzymes. J. Physiol..

[20] De Rosa A., Monaco M.L., Capasso M., Forestieri P., Pilone V., Nardelli C., Buono P., Daniele A. (2013). Adiponectin oligomers as potential indicators of adipose tissue improvement in obese subjects. Eur. J. Endocrinol..

[21] Godoy–Matos A.F., Bahia L.R., Domingues R.C., Sicuro F., Tambascia M., Geloneze B., Kraemer–Aguiar L.G., Bouskela E. (2010). Adiponectin is related to intramyocellular lipid content in non–diabetic adults. Endocrinol. Invest. J..

[22] Yamamoto S., Matsushita Y., Nakagawa T., Hayashi T., Noda M., Mizoue T. (2014). Circulating adiponectin levels and risk of type 2 diabetes in the Japanese. Nutr. Diabetes.

[23] Gomez–Merino D., Drogou C., Guezennec C.Y., Chennaoui M. (2007). Effects of chronic exercise on cytokine production in white adipose tissue and skeletal muscle of rats. Cytokine.

[24] Simpson K.A., Singh M.A. (2008). Effects of exercise on adiponectin: A systematic review. Obesity (Silver Spring).

[25] Esfahani M., Movahedian A., Baranchi M., Goodarzi MT. (2015). Adiponectin: An adipokine with protective features against metabolic syndrome. Iran. J. Basic Med. Sci..

[26] Nunes R., Silva P., Alves J., Stefani G., Petry M., Rhoden C., Dal Lago P., Schneider C.D. (2013). Effects of resistance training associated with whey protein supplementation on liver and kidney biomarkers in rats. Appl. Nutr. Metab..

[27] Gerosa–Neto J., Antunes B.M.M., Campos E.Z., Rodrigues J., Ferrari G.D., Rosa Neto J.C., Bueno C.R., Lira F.S. (2016). Impact of long-term high-intensity interval and moderate-intensity continuous training on subclinical inflammation in overweight/obese adults. J. Exerc. Rehabil..

[28] Ahmadizad S., Haghighi A.H., Hamedinia M.R. (2007). Effects of resistance versus endurance training on serum adiponectin and insulin resistance index. Eur. J. Endocrinol..

[29] Sirico F., Bianco A., D’Alicandro G., Castaldo C., Montagnani S., Spera R., Di Meglio F., Nurzynska D. (2018). Effects of physical exercise on adiponectin, leptin, and inflammatory markers in childhood obesity: Systematic review and meta–analysis. Child. Obes..

[30] Stanford K.I., Goodyear L.J. (2016). Exercise regulation of adipose tissue. Adipocyte.

[31] Martinez–Huenchullan S.F., Maharjan B.R., Williams P.F., Tam C.S., Mclennan S.V., Twigg S.M. (2018). Skeletal muscle adiponectin induction depends on diet, muscle type/activity, and exercise modality in C57BL/6 mice. Physiol. Rep..

[32] Nakagawa Y., Hattori M. (2017). Intramyocellular lipids of muscle type in athletes of different sport discipline. Open. Acc. J. Ports. Med..

[33] Lee S., Kwak H.B. (2014). Effects of interventions on adiponectin and adiponectin receptors. J. Exerc. Rehabil..

